# The HSA21 gene *EURL/C21ORF91* controls neurogenesis within the cerebral cortex and is implicated in the pathogenesis of Down Syndrome

**DOI:** 10.1038/srep29514

**Published:** 2016-07-11

**Authors:** Shan Shan Li, Zhengdong Qu, Matilda Haas, Linh Ngo, You Jeong Heo, Hyo Jung Kang, Joanne Maria Britto, Hayley Daniella Cullen, Hannah Kate Vanyai, Seong-Seng Tan, Tailoi Chan-Ling, Jenny Margaret Gunnersen, Julian Ik-Tsen Heng

**Affiliations:** 1 EMBL Australia, The Australian Regenerative Medicine Institute, Monash University, Clayton, Victoria 3800, Australia; 2 The Harry Perkins Institute of Medical Research, QEII Medical Centre, Nedlands and Centre for Medical Research, The University of Western Australia, Crawley, Western Australia, 6009, Australia; 3 Department of Life Science, Chung-Ang University, Seoul, Korea; 4 Department of Neurobiology, Yale School of Medicine, New Haven, CT 06510, USA; 5 The Florey Institute of Neuroscience and Mental Health, Parkville, Victoria 3010, Australia; 6 Discipline of Anatomy, School of Medical Sciences, Bosch Institute, The University of Sydney, Sydney, New South Wales, Australia; 7 The Anatomy and Neuroscience Department, University of Melbourne, Parkville, Victoria 3010, Australia

## Abstract

Copy number variations to chromosome 21 (HSA21) cause intellectual disability and Down Syndrome, but our understanding of the HSA21 genetic factors which contribute to fetal brain development remains incomplete. Here, we focussed on the neurodevelopmental functions for *EURL* (also known as *C21ORF91*, Refseq Gene ID:54149), a protein-coding gene at the centromeric boundary of the Down Syndrome Critical Region (DSCR) of HSA21. We report that EURL is expressed during human and mouse cerebral cortex development, and we report that alterations to *EURL* mRNA levels within the human brain underlie Down Syndrome. Our gene perturbation studies in mice demonstrate that disruptions to *Eurl* impair progenitor proliferation and neuronal differentiation. Also, we find that disruptions to *Eurl* impair the long-term positioning and dendritic spine densities of cortical projection neurons. We provide evidence that EURL interacts with the coiled-coil domain-containing protein CCDC85B so as to modulate β-catenin levels in cells. Further, we utilised a fluorescent reporter (8xTOPFLASHd2EGFP) to demonstrate that disruptions to *Eurl* alter β-catenin signalling *in vitro* as well as *in vivo*. Together, these studies highlight EURL as an important new player in neuronal development that is likely to impact on the neuropathogenesis of HSA21-related disorders including Down Syndrome.

The development of the cerebral cortex relies on the timely production of neurons from local pools of progenitor cells. These new neurons then undergo migration to position themselves within the growing tissue before establishing appropriate connections with other cells so as to form functional circuits. Failure in these early stages of brain development can lead to intellectual disability, cognitive impairment and epilepsy^1^,^2^,^3^. Abnormal brain development can arise through genetic abnormalities during fetal development, such as copy number variations to chromosome 21 (HSA21). In humans, Down Syndrome (DS) is the most commonly diagnosed HSA21 aneuploidy disorder, with an incidence of 1 in 700 live births^4^,^5^. Intellectual disability is an invariant diagnosis of DS, and arises as a result of defective fetal brain development. Indeed, patients diagnosed with DS show reduced brain volume^6^, defective cortical organisation and displacement of neurons within the cerebral cortex^7^ as well as altered neuronal connectivity during fetal development^8^,^9^,^10^,^11^ and epilepsy^12^,^13^.

It is recognised that HSA21-related aneuploidy disorders are a consequence of an improper dosage of triplicated genes, a concept which can be referred to as the “gene dosage hypothesis”^4^,^5^. Recent focus on a HSA21 region encompassing 21q21–21q22.3 described as the Down Syndrome Critical Region (DCSR), has established important functional roles for genes lying within the DSCR (such as *DSCAM*, *DYRK1A* and *OLIG2*) during brain development^14^,^15^,^16^,^17^. However, genetic association studies of HSA21-related aneuploidy disorders point to additional candidate genes beyond the DSCR which are important for neuronal development, and whose functions are likely to be relevant to the neurobiology of intellectual disability^18^,^19^,^20^. *EURL* (also known as C21ORF91), a protein-coding gene which lies at the centromeric boundary of the DSCR (within 21q21.1), is one such candidate gene which is poorly characterised.

Originally identified as a gene expressed in undifferentiated retina and lens of chick embryos^21^, *EURL* was observed to be triplicated in HSA21-related aneuploidy states in humans^18^,^19^,^20^. For example, in Down Syndrome patients with rare segmental HSA21 trisomies, it has been suggested that gene dosage imbalances of a locus comprising *EURL* may account for their intellectual disability^18^. Furthermore, studies of lymphoblastic cells derived from Down Syndrome patients show that *EURL* mRNA expression is elevated^22^,^23^. Interestingly, a study by Rost and colleagues described an intellectually disabled patient with a partial tetrasomy of HSA21 (comprising *EURL*), but lacking the clinical features of Down syndrome^19^. Similarly, Slavotinek and colleagues described a male infant with microcephaly which harboured a partial tetrasomy 21 (also comprising *EURL*) which did not encompass the Down Syndrome Critical Region^20^. Taken together, these observations suggest that perturbations to *EURL* may be a contributing genetic factor in the neurobiology of brain growth and intellectual disability.

Here, we explore the expression pattern and the functions of EURL in cerebral cortex development. We report that EURL is expressed within the developing central nervous system (CNS) of mice and humans, and is detected in neural progenitor cells and postmitotic neurons of the embryonic cerebral cortex. We perform a series of gene perturbation studies with mice and report that knockdown or overexpression of *Eurl* influences neuroprogenitor proliferation, and neuronal differentiation. We further explore the downstream molecular mechanism for EURL and provide evidence that it is a novel modulator of β-catenin signalling during fetal brain development. Finally, we provide evidence that *EURL* mRNA expression within the brains of Down Syndrome (Trisomy21) patients is altered. Altogether, we highlight the concentration-dependent effects of EURL on neuronal development within the immature cerebral cortex, and suggest *EURL* as a contributory gene to the neurodevelopmental disorders arising from HSA21 aneuploidies in infants.

## Results

### EURL is expressed during cortical development

**Figure 1:**
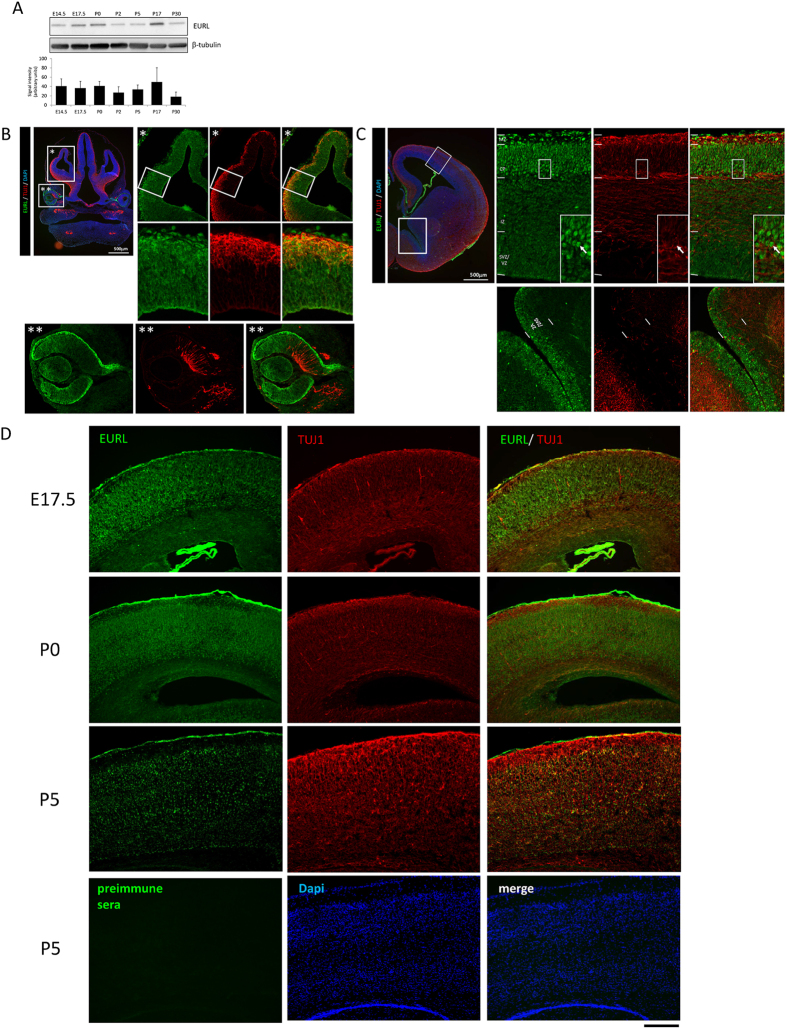
Expression of EURL during mouse brain development. (**A**) Western blotting analysis of EURL protein levels (represented as an immunoreactive band of approximately 34kDa) in brain lysates over the course of mouse brain development, from embryonic (E14.5, E17.5) to postnatal (P0, P2, P5, P17, P30). Signals for β-tubulin (approximately 50kDa in size) serve as loading control. (**B**) Immunostaining reveals prominent EURL reactivity (green signal) within the dorsal telencephalon (region magnified in “*”) as well as the developing retina and lens (region magnified in “**”). Double immunostaining with the early neuronal marker TUJ1 (red) reveals EURL immunoreactivity in neural and non-neural cells. (**C**) Within the E14.5 dorsal telencephalon, EURL immunoreactivity is strong in posmitotic cells of the cortical plate (CP) and marginal zone (MZ), with a weaker signal detected in cells of the intermediate zone (IZ) and germinal subventricular/ventricular zone (SVZ/VZ). A CP neuron is represented with an arrow in the boxed insert. (**D**) At late embryonic (E17.5) stages, EURL immunoreactivity persists in CP neurons, as well as in postnatal (P0, P5) brains. The immunostaining signals detected with the EURL antibody are not evident when a parallel experiment is conducted with preimmune serum. Scale bar, 200 μm.

We performed Western blotting to survey EURL expression during mouse brain development (Fig. 1A). We found that Eurl is detected in brain lysates at all timepoints, from fetal embryonic day (E)14.5, E17.5, as well as in newborn (P0) tissues through to juvenile brains (P30) (n = 2–3 samples per timepoint of protein extraction). We also performed a series of immunostaining studies to identify the sites of EURL immunoreactivity within the CNS, with particular focus on the forebrain. Within the embryonic (E12.5) cortex, EURL immunoreactivity was evident within the germinal ventricular zone (VZ) and in cells of the cortical plate (CP), which express the early neuronal marker TUJ1 (Fig. 1B). EURL immunoreactivity was also detected in the retinal pigment epithelium and lens (Fig. 1B*) consistent with earlier findings (21), while TUJI expression was evident at the vitreal surface of the neuroretina. Within the E14.5 forebrain, *Eurl* gene expression is evident within the embryonic cortex (Supplementary Fig. 1B)^24^, and EURL immunoreactivity was conspicuous in postmitotic Cortical Plate (CP) neurons of the embryonic cerebral cortex which also express TUJ1, while a moderate signal was also detected in the germinal VZ and the intermediate zone (IZ) (Fig. 1C). In the ganglionic eminences of the ventral telencephalon, Eurl immunoreactivity was observed in cells of the VZ which give rise to interneurons of the cerebral cortex. EURL brain immunoreactivity was also detected at late gestational (E17.5), as well as at newborn (P0) and postnatal (P5) stages (Fig. 1D). Parallel immunostaining experiments performed with preimmune serum did not yield a signal.

**Figure 2:**
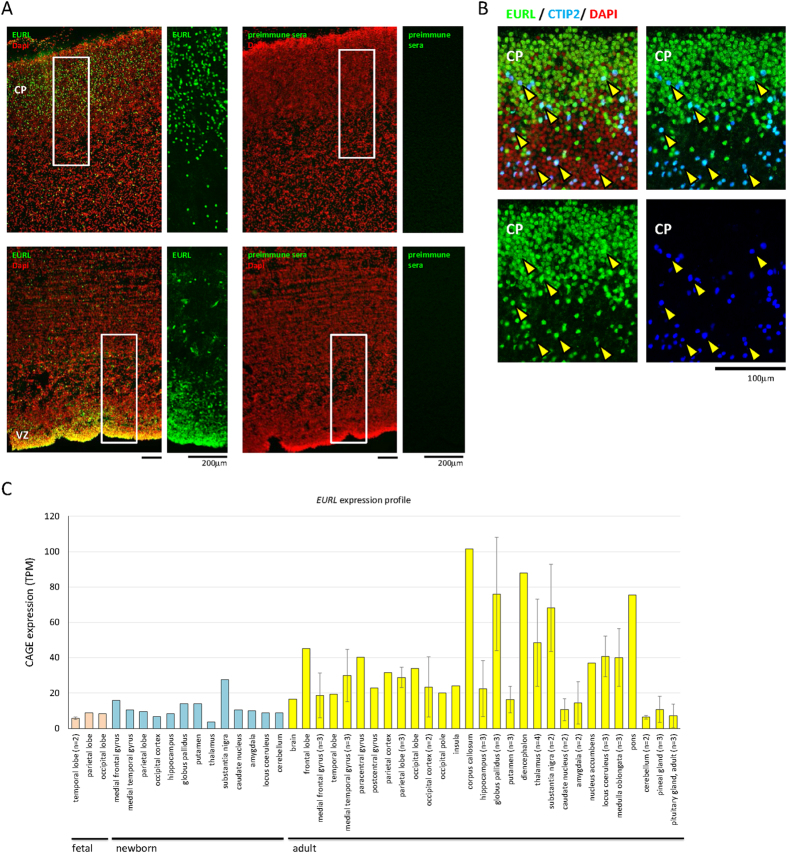
Immunostaining for EURL expression within the human fetal brain. (**A**) Immunostaining of human fetal cerebral cortex at 16 weeks post conception (GW16) revealed prominent EURL immunoreactivity in cells of the germinal ventricular zone (VZ) and the developing cortical plate (CP). Parallel experiments with preimmune serum did not elicit a signal. (**B**) Co-labelling studies demonstrate that EURL is expressed in projection neurons marked with CTIP2 (arrowheads) Nuclei are stained with DAPI. (**C**) A survey of *EURL* mRNA expression in human brain tissue samples from fetal (pink bars), newborn (light blue) and adult (yellow bars) generated through Capped Analysis of Gene Expression (CAGE) in FANTOM5^25^. Quantitative data was normalized across libraries and expressed as Tags Per Million (TPM) mapped reads in a given CAGE library. Where multiple samples were available from a given tissue, data is plotted as an average ± standard deviation. As a guide, expression levels at 10 TPM reflect approximately 3 copies of a given transcript in a cell^25^.

To characterise EURL expression during human fetal cortical development, we performed immunostaining for EURL on sections of fetal (GW16) cortex. We observed prominent EURL immunoreactivity in cortical cells of the VZ (Fig. 2A). We also detected prominent EURL immunoreactivity within cells of the Cortical Plate (CP), including a subpopulation of neurons immunolabelled with CTIP2 (Fig. 2B). We surveyed *EURL* gene expression in the data collection FANTOM5^25^ to find that it is expressed in cells of the human fetal, newborn and adult brain (Fig. 2C). Together, these results reveal a dynamic expression pattern for *EURL* in mouse and human brain, and suggest that changes to its temporal expression levels may impact on brain development.

### *EURL* expression in the brain is altered in Down Syndrome

**Figure 3:**
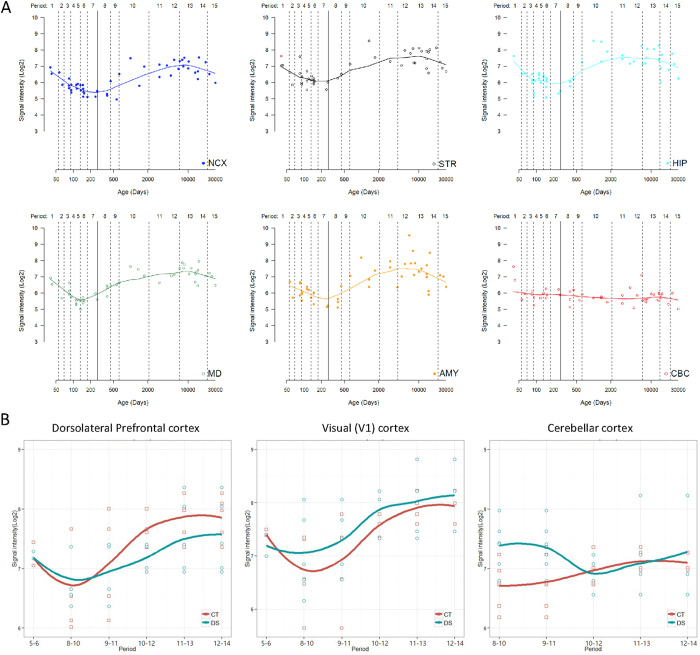
Dynamic expression of *EURL*/*C21ORF91* in the human brain. (**A**) Temporal analysis of *EURL*/*C21ORF91* mRNA in the human brain reveals a dynamic expression pattern within the neocortex (NCX), striatum (STR), hippocampus (HIP), mediodorsal nucleus of the thalamus (MD), amygdala (AMY); while expression levels within the cerebellar cortex (CBC) are relatively stable throughout all ages tested. The solid line represents birth (defined as 280 days). The line for each region represent media values, and are generated by LOESS regression method using “ggplot2” package, as previously described^45^. (**B**) Analysis of *EURL*/*C21ORF91* mRNA expression in the dorsolateral prefrontal cortex (DFC), primary visual cortex (V1C) and cerebellar cortex (CBC) reveals different levels of gene expression between Down Syndrome (DS, in green) patients and age-matched controls (CT, in red). One-tailed *t*-tests were applied to gene expression datasets for DFC, V1C and CBC to investigate potential differences in gene expression levels, as described. To account for the variabilities in tissues collected for gene expression studies, a “sliding window” approach was adopted in which mRNA signals from closely matched ages of tissue samples were grouped (see Methods for further details). Age ranges for Periods 5..6 (postconception week 14pwc, 15pcw, 16pcw, 17pcw), 8..10 (1 month old (mo), 4mo, 6mo, 9mo, 10mo, 1 year (yr), 2yr, 3yr), 9..11 (6mo, 9mo, 10mo, 1yr, 2yr, 3yr, 8yr, 10yr), 10..12 (2yr, 3yr, 8yr, 10yr, 13yr, 15yr, 18yr, 19yr), 11..13 (10yr, 13yr, 15yr, 18yr, 19yr, 22yr, 37yr, 39yr), 12..14 (13yr, 15yr, 18yr, 19yr, 22yr, 37yr, 39yr, 40yr, 42yr).

We sought to substantiate the notion that alterations to *EURL* expression might underlie HSA21-related neurological disorders. We investigated longitudinal gene expression analysis on human brain tissue samples^26^ and found that *EURL* expression in neocortex (NCX), striatum (STR), hippocampus (HIP), mediodorsal nucleus of the thalamus (MD) and amygdala (AMY) followed a periodicity of gene expression, with mRNA levels decreasing during fetal development followed by a gradual increase following birth before a final, gradual decrease by 10,000 days (approximately 27 years) (Fig. 3A). These trends are consistent with the gene expression patterns for *EURL* observed in FANTOM5 in which we find moderate increases in levels from fetal brain to postnatal to adult (Fig. 2C)^25^. In contrast, *EURL* expression in the cerebellar cortex (CBC) remained relatively constant, with a trend towards a slight decrease (Fig. 3A). It is also noteworthy that the patterns of *EURL* expression within these human cortical regions DFC and V1C are reminiscent of Eurl protein expression studies with mouse brain lysates (Fig. 1A).

We were particularly interested in the gene expression patterns for *EURL* within the dorsolateral prefrontal cortex (DFC), primary visual cortex (V1C) and cerebellar cortex (CBC) because of the importance of these regions for cognition (DFC), sight (V1C) and coordinating movement (CBC), respectively (Fig. 3B). In these three regions of the brain, we observed relatively low expression levels for *EURL* within the period comprising tissues isolated from birth-to-6 years of age (denoted as “period 8–10”), before mRNA levels steadily increased with age and throughout life. Finally, we examined *EURL* mRNA expression levels in patients with Down Syndrome in which trisomy 21 was confirmed (see Methods). When compared with controls, we observed a significant difference in the abundance of *EURL* mRNA levels within all three brain regions (DFC, V1C, CBC) analysed (Fig. 3B). Furthermore, we found significant differences in the levels of *EURL* gene expression within the DFC in samples from early postnatal life towards adulthood (“period 10..12” representing tissues collected from 1year to 20years, p = 0.006, n = 4, *t*-test one-tailed), as well as in the CBC during early childhood (period “8..10” representing tissues collected from birth to 6 years, p = 0.024, n = 6, *t*-test one-tailed). Thus, we find that the expression of *EURL* during brain development is dynamic, and also that mRNA levels are disrupted in Down Syndrome.

### Forced expression or knockdown of *Eurl* disrupts neurogenesis in the cerebral cortex

**Figure 4:**
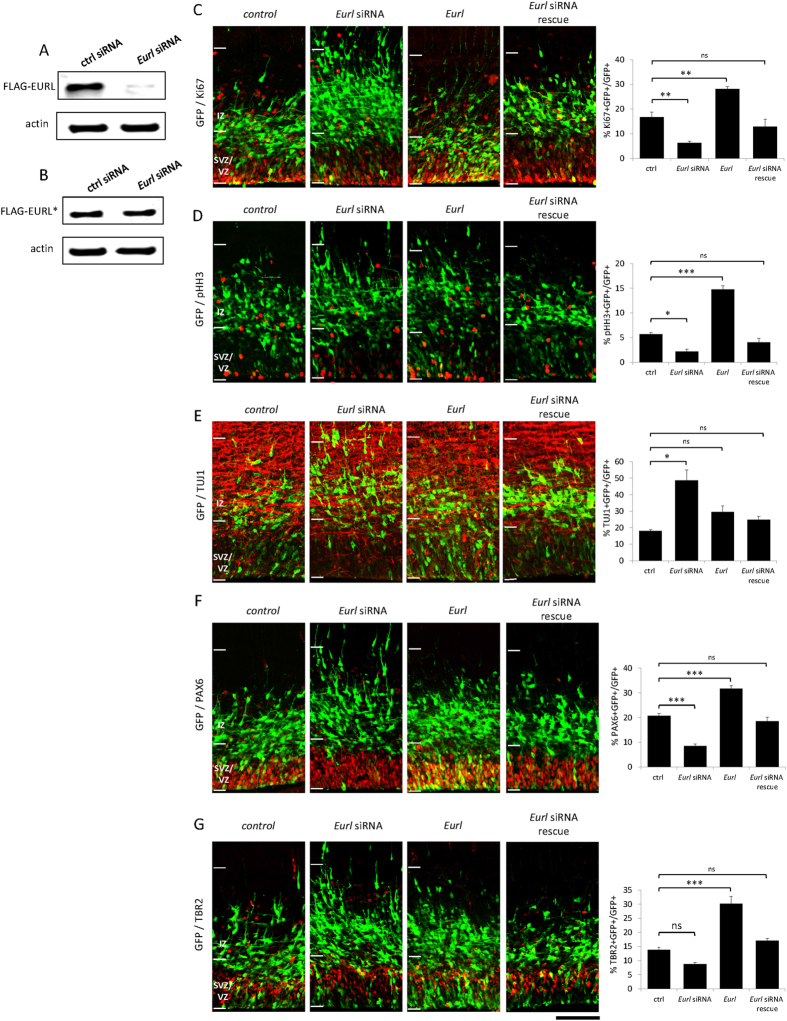
Alterations to *Eurl* expression disrupt neuroprogenitor proliferation and neurodifferentiation within the embryonic cerebral cortex. Specificity of knockdown using *Eurl* siRNAs in Western blotting assays. (**A,B**) *Eurl* siRNAs knockdown FLAG-EURL expression (represented as a 30kDa band) in transiently transfected P19 mouse embryocarcinoma cells, but does not knock down a FLAG-EURL* cDNA construct which is refractory to silencing owing to engineered silent mutations (see methods). Beta-actin (a 42kDa immunoreactive band) immunoreactivity was used as a loading control. *In utero* electroporation experiments in which E14.5-born cortical cells are transduced with GFP vector, together with *Eurl* siRNAs to knockdown expression; or to force express *Eurl* by introducing an expression construct. Brain sections were processed for fluorescence immunostaining to reveal GFP signal, as well as two markers of cell proliferation (Ki67, pHH3). Disruptions to *Eurl* resulted in significant changes to GFP+ cells co-labelled with Ki67 (F_3,10_ = 34, p < 0.0001) (**C**), pHH3 (F_3,11_ = 76, p < 0.0001) (**D**); or TUJ1 expression (F_3,15_ = 12, p = 0.0003) (**E**). (**F,G**) Knockdown of *Eurl* resulted in a significant reduction in GFP + cells which co-label with the radial glial progenitor marker PAX6, while forced expression led to a significant increase (F_3,10_ = 54, p < 0.0001). The *Eurl* siRNA-mediated effects on PAX6 expression could be abrogated by co-delivery with an expression construct which is refractory to silencing (see Methods). Disruptions to *Eurl* also led to changes in TBR2 immunoreactivity of GFP+ cells (F_3,11_ = 43, p < 0.0001). At least 600–800 cells were counted from 3–4 brains per condition, per treatment. One way ANOVA followed by Bonferroni’s posthoc test corrected for multiple testing. *P < 0.05, **P < 0.01, ***P < 0.001, while ns indicates comparison is not significant. Scale bar, 100 μm.

We wanted to determine whether disruptions to *Eurl* gene expression affected cerebral cortical development. We performed *in utero* electroporation experiments on time-mated E14.5 pregnant mice, delivering a pool of siRNAs targeting *Eurl* coding sequence as well as the 3′UTR (Fig. 4A and see Methods) together with a mammalian reporter construct encoding green fluorescent protein (GFP) into cells of the dorsal cortical VZ. Parallel experiments were performed with a bi-cistronic construct encoding *Eurl* and GFP to investigate the effects of forced expression on VZ cells. Control experiments were performed with non-targeting siRNAs together with a GFP construct. Thirty-six hours post electroporation, brain samples were collected and processed for immunostaining experiments on cryo-sectioned tissue. As shown in Fig. 4C,D, we found that knockdown of *Eurl* led to a decrease in GFP+ cells that co-label with the proliferation markers Ki67 and pHH3 (phosphorylated histone H3, a marker of cell mitosis), thereby suggesting a disruption to cortical progenitor proliferation and premature neurogenesis. In support of this notion, we also observed a concomitant increase in the proportion of neurons which co-label with the early neuronal marker TUJ1 (Fig. 4E). In contrast, however, forced expression of *Eurl* led to a significant increase in the proportion of GFP+ cells which co-label with Ki67 and pHH3, again suggesting that balanced levels of *Eurl* influence cortical progenitor proliferation, and the number of young neurons with TUJ1 co-expression was not significantly altered. To account for the specificity of the effects of knockdown, we performed rescue experiments in which *Eurl* siRNA-treated cells were co-electroporated with an *Eurl* expression construct encoding silent mutations which renders it refractory to silencing (Fig. 4B). We found that the alterations in Ki67, pHH3 and TUJ1 expression in *Eurl*-deficient cells could be restored by co-delivery of *Eurl* (Fig. 4C–E).

To further clarify the effects of *Eurl* perturbations on neuroprogenitor cells within the embryonic cerebral cortex, we performed immunostaining to discriminate between PAX6-expressing radial glial progenitors (which divide to produce progenitors and neurons) and TBR2-expression intermediate progenitors (which divide symmetrically to produce two daughter neurons) (reviewed in^2^,^27^) (Fig. 4F,G). Our results show that knockdown of *Eurl* led to significant reductions in the proportions of PAX6+ radial glial progenitors, and this RNAi effect could be restored by co-delivery of *Eurl* (Fig. 4F). Knockdown of *Eurl* did not lead to a significant reduction in TBR2+ intermediate progenitor cells. On the other hand, forced expression of *Eurl* led to a significant increase in both progenitor populations (Fig. 4G). Together, these results indicate that disruptions to *Eurl* alter the proliferation of both radial glial progenitors and intermediate progenitors in a concentration-sensitive manner.

### Changes to *Eurl* expression alter the long-term positioning and dendritic spine densities of cerebral cortical neurons

**Figure 5:**
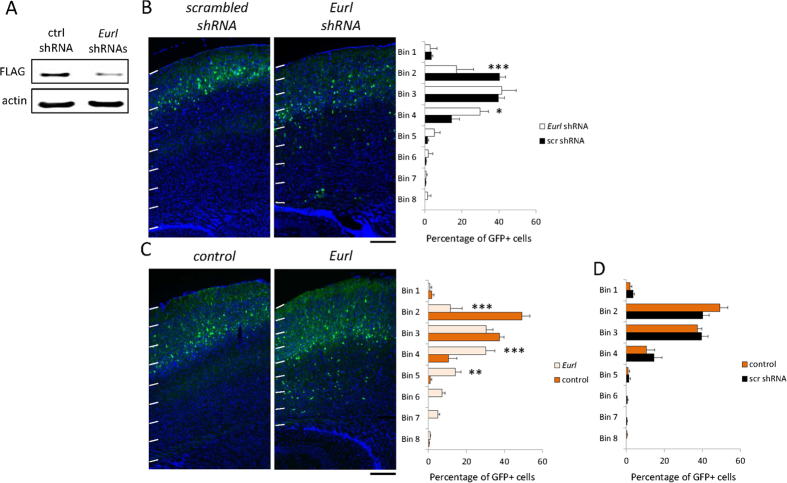
Knockdown as well as over-expression of *Eurl* alters the long-term positioning of cortical projection neurons within the postnatal (P17) cerebral cortex. (**A**) Treatment with *Eurl* shRNAs leads to suppression of FLAG-EURL levels in transiently transfected P19 cells, visualised by Western blotting. (**B**) Treatment with *Eurl* shRNAs impaired the positioning of E14.5-born, GFP-labelled cortical projection neurons, as determined by the proportion of cells within the cortex which is divided into 8 arbitrary bins (F_7,32_ = 5.5, P = 0.0003). (**C**) Forced expression of *Eurl* also disrupted the long-term positioning of E14.5-born cortical projection neurons (F_7,32_ = 5.5, P < 0.0001). (**D**) The migration profile of cells treated with control shRNA (scr) vector, or GFP-only control vector was not significantly different (F_7,32_ = 2.0, P = 0.0838). At least 700 cells were counted from 3 brains per condition. Two-way ANOVA analysis was performed in each case, followed by a Bonferroni posthoc test with multiple correction testing, *P < 0.05, **P < 0.01, ***P < 0.001. Scale bars, 200 μm.

We next studied the effects of *Eurl* gene disruption on the maturation of cortical projection neurons. For prolonged gene suppression by RNA interference, we introduced an shRNA construct comprising an *Eurl* targeting hairpin sequence (Fig. 5A and Supplementary Fig. 1C) together with a GFP expression cassette into cortical cells of E14.5 embryos. Control experiments were performed with a previously characterised, non-targeting shRNA sequence^28^. We performed *in utero* electroporation and then collected brain tissue for analysis at postnatal day P17, a time when neurons have achieved their appropriate positioning. Our results show that control GFP-labelled cells are distributed within the upper regions of the P17 cortex, while treatment with *Eurl* shRNA led to a significant impairment in the positioning of GFP-labelled cells, with a significant proportion of cells lying deep within the cortex (Fig. 5B). Interestingly, we found that forced expression of *Eurl* also altered cell positioning within the postnatal cortex (Fig. 5C). We additionally confirmed that knockdown or overexpression of Eurl did not significantly disrupt neuronal differentiation as judged by co-expression of NeuN (Supplementary Fig. 1D), and also that the positioning of GFP+ labelled cells harbouring the non-targeting shRNA sequence was not significantly different between control conditions (Fig. 5D).

**Figure 6:**
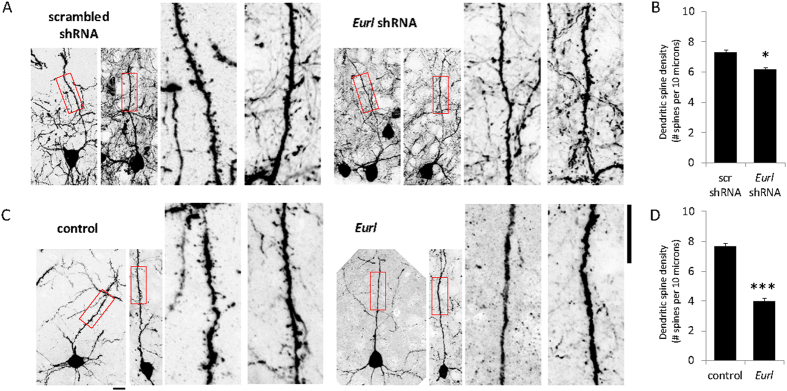
Disruptions to *Eurl* expression lead to a decrease in dendritic spine densities of layer II/III projection neurons within the P17 cortex. (**A,B**) High-power confocal imaging of GFP-labelled neurons within layer II/III of the cortex to quantify dendritic spine densities. Treatment with *Eurl* shRNAs led to a decrease in dendritic spine density (p = 0.0228, Two-tailed *t*-test) (n = 27 *scr* shRNA-treated neurons and n = 19 *Eurl* shRNA-treated neurons). (**C,D**) Forced expression of *Eurl* led to a decrease in dendritic spine densities compared with control treatment (p < 0.001, Two-tailed *t*-test) (n = 40 control (GFP vector only) treated neurons and n = 27 neurons treated with *Eurl* bicistronic vector encoding GFP as well). The dendritic spine densities of cells from control shRNA-treatment were not significantly different to treatment with GFP-only control vector (p = 0.2293, Two-tailed *t*-test; graph not shown). Scale bars, 10 μm.

Next, we determined whether disrupted *Eurl* expression might affect the dendritic spine development of post-migratory cortical projection neurons. We performed high-magnification confocal microscopy imaging of GFP-labelled neurons within layer II/III of the cortex from each of the treatment conditions to study their dendritic spine densities. Our results show that treatment with *Eurl* shRNAs led to a significant reduction in dendritic spine densities along the primary apical dendrite, compared to treatment with a non-targeting shRNA (n = 27 and 19 for non-targeting scr shRNA treatment versus *Eurl* shRNA-treated dendrites, respectively; p = 0.0228, Two-tailed *t*-test). Similarly, forced expression of *Eurl* also led to a decrease in spine densities compared with control (GFP only) treatment (n = 40 and 27 for control (GFP only) treatment versus Eurl vector-treated neurons, respectively; p < 0.001, Two-tailed *t*-test). The dendritic spine densities of cells from control (non-targeting) shRNA-treatment were not significantly different to treatment with control (GFP only) vector (p = 0.2293, Two-tailed *t*-test) (Fig. 6). Therefore, perturbations to *Eurl* expression alter the long-term positioning and dendritic spine densities of cortical projection neurons within the postnatal mouse cerebral cortex.

### *Eurl* interacts with Ccdc85b and modulates canonical β-catenin signalling

**Figure 7:**
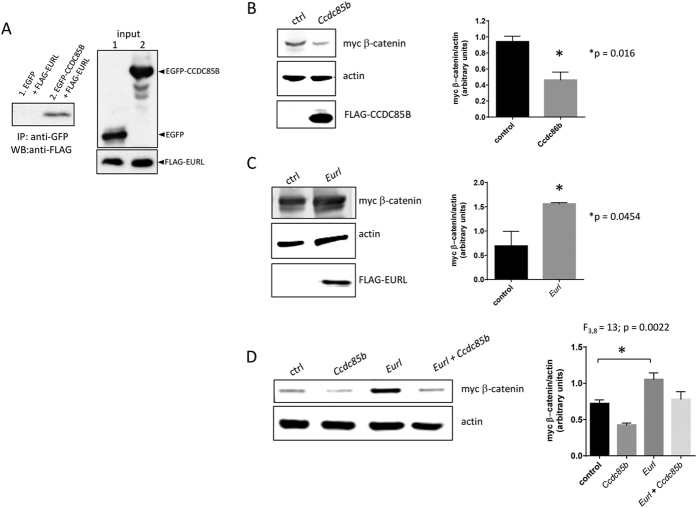
EURL interacts with CCDC86B *in vitro* and influences β-catenin levels in HEKT293T cells. (**A**) Co-immunoprecipitation experiments with a FLAG-tagged EURL polypeptide confirm an interaction with GFP-CCDC85B fusion protein, and not GFP alone. Input lanes confirm all proteins are expressed in each condition tested. (**B**) In HEK293T cells, forced expression of CCDC85B leads to a reduction in myc β-catenin levels in transient transfection assays. (**C**) Forced expression of *Eurl* leads to an increase in myc β-catenin levels. (**D**) Forced expression of *Ccdc85b* suppresses myc β-catenin, but co-delivery of *Eurl* abrogates this effect. Quantification of immunoblotting signals was performed on three independent experiments.

To explore the potential molecular mechanism through which Eurl exerts its cellular effects, we searched the on-line resource BioGRID^29^ and identified CCDC85B/DIPA as a putative interacting partner among several candidates. We were interested to study this putative interacting partner to Eurl because Ccdc85b was recently reported to be important for nervous system development^30^, and was previously reported to mediate β-catenin signalling through a mechanism involving protein stabilisation^31^. Also, we find that *CCDC85B* is expressed in the brain, and its expression levels are also disturbed in Down Syndrome (Supplementary Fig. 2). To establish their protein-protein interaction, we performed a co-immunoprecipitation experiment with heterologous cells. We found that FLAG-tagged Eurl is co-immunoprecipitated by a GFP-Ccdc85b fusion protein, but not with GFP alone (Fig. 7A). Our attempts to generate an antibody to study native Ccdc85b expression and protein-protein interaction *in vivo* have so far been unsuccessful (data not shown).

Next, we explored the potential for Eurl and Ccdc85b to signal cooperatively in cells. It was previously reported that CCDC85B induces the degradation of β-catenin protein as a molecular mechanism to suppress cell proliferation^31^, hence we wanted to determine if Eurl could modulate this activity of Ccdc85b. We performed transient transfection experiments in which we delivered expression constructs for *Eurl* and *Ccdc85b* together with an expression construct encoding myc-tagged β-catenin, and then examined the levels of myc-tagged β-catenin by Western blotting. As shown, transfection of *Ccdc85b* resulted in a decrease in myc-tagged β-catenin in transiently transfected HEK293T cells (Fig. 7B), a finding consistent with the study by Iwai and colleagues^31^. In contrast, transfection of *Eurl* led to a significant increase in myc-tagged β-catenin levels in this assay (Fig. 7C). Interestingly, Ccdc85b-induced reduction in myc-tagged β-catenin levels in HEK293T cells can be curtailed by co-transfection of *Eurl*, suggesting that the presence of Eurl affects the capacity for Ccdc85b to suppress β-catenin protein levels in this assay (Fig. 7D).

**Figure 8:**
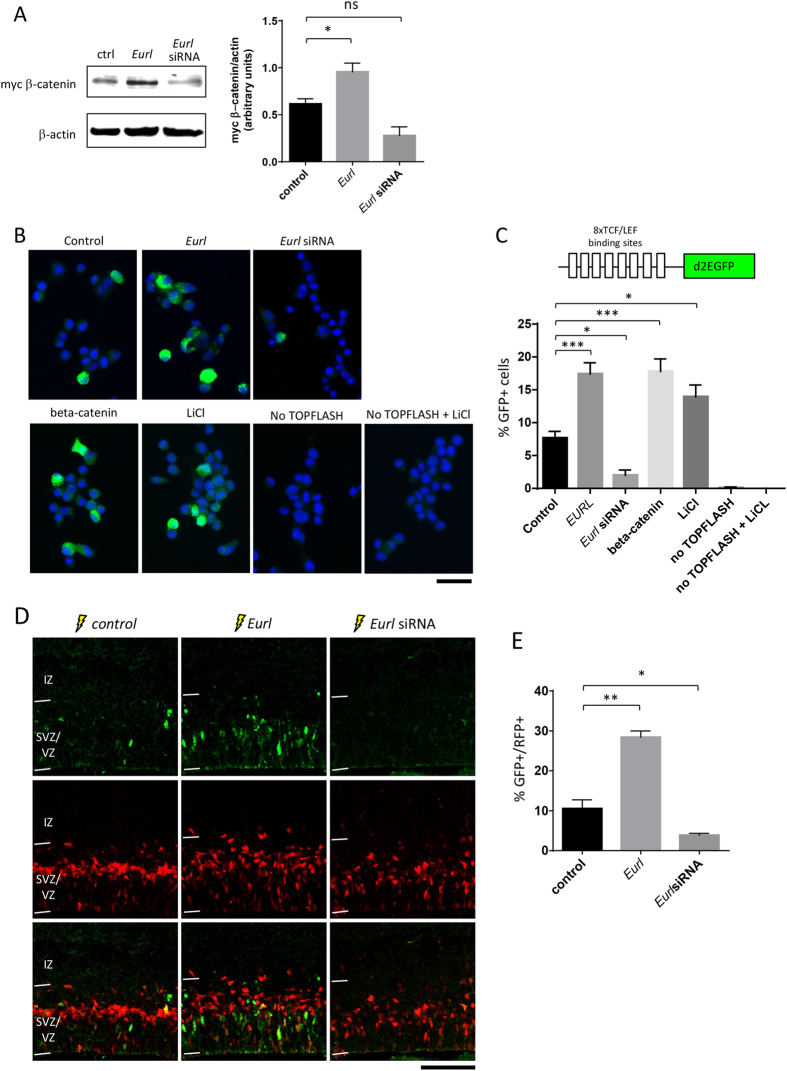
Effects of *Eurl* gene disruption on β-catenin signalling *in vitro* and *in vivo*. (**A**) Forced expression of *Eurl* led to an increase in myc β-catenin levels, while knockdown with *Eurl* siRNAs lead to a trend towards a decrease (F_2,6_ = 18, P = 0.0031). Quantification of immunoblotting signals were performed on three independent experiments. (**B,C**) Neuro2a cells were transiently transfected with a reporter of β-catenin signalling (known as 8XTOPFLASHd2EGFP) together with *Eurl* expression construct, *Eurl* siRNAs, or beta-catenin expression construct as a positive control for reporter activity. An additional positive control was conducted in which 8XTOPFLASHd2EGFP transfected cells were treated with 50 mM lithium chloride (LiCl) for 14 hours prior to data collection. As negative controls, cells were transfected with empty expression construct and control (non-targeting) siRNAs but not 8XTOPFLASHd2EGFP reporter, followed by treatment with or without 50 mM LiCl. Forty-eight hours post transfection, cells were fixed and GFP-expressing cells were quantified to reveal a significant interaction between *Eurl* gene disruption and GFP epifluorescence (F_6,14_ = 40, P < 0.0001, One-WAY ANOVA, n > 900 cells from triplicate experiments per condition). Forced expression of *Eurl* led to an increase in the proportion of GFP-expressing cells, while knockdown led to a significant decrease in GFP-expressing cells. (**D,E**) *Eurl* gene disruption alters β-catenin signalling within the embryonic cerebral cortex. E14.5 cortices were electroporated with 8XTOPFLASHd2EGFP vector, together with mRFP vector to mark electroproated cells. Compared to control (empty vector) treatment, forced expression of *Eurl* resulted in an increase in GFP-expressing cells, while treatment with *Eurl* siRNAs resulted in a decrease in GFP-expressing cells (F_2,15_ = 35, P < 0.0001, One-WAY ANOVA followed by a Bonferroni posthoc test with multiple correction testing, *P < 0.05, **P < 0.01, ***P < 0.001). At least 700 cells were counted from 3 brains per condition. Scale bars, 50 μm (**B**), 100 μm (**D**).

We extended these *in vitro* studies to determine how disruptions to *Eurl* gene expression might influence β-catenin signalling in Neuro2a cells, a mouse neural cell line. Consistent with our findings in HEK293T cells, we observed that forced expression of *Eurl* resulted in an elevation of myc-tagged β-catenin levels in Neuro2a cells (Fig. 8A), while knockdown of *Eurl* with targeting siRNAs led to reduced levels compared to control (non-targeting) siRNA treatment, although the latter effect was not statistically significant, despite a trend. We next developed a functional assay to determine how alterations to *Eurl* expression might alter β-catenin signalling, using a 8xTOPFLASHd2EGFP reporter construct^32^. This plasmid reporter comprises multiple copies of Tcf/Lef1 binding sites upstream of a destabilised EGFP cDNA encoding a green fluorescent polypeptide (with a short half-life of 20 minutes), and reflects Wnt/β-catenin signalling activity. As shown in Fig. 8B,C, we found that forced expression of *Eurl* resulted in a significant increase in the number of GFP+ cells compared with control treatment. In contrast, treatment with *Eurl* siRNAs led to a significant decrease in GFP+ cells. As positive controls for this experiment, we performed parallel experiments in which cells were transfected with β-catenin expression construct, or stimulated with lithium chloride (an inhibitor of GSK3 signalling)^33^, and we observed a significant increase in GFP+ cells for both these treatments. We did not observe GFP+ cells in negative control experiments in which cells were not transfected with TOPFLASH reporter construct, or when cells did not receive TOPFLASH reporter but was treated with lithium chloride.

Guided by our results with Neuro2a cells, we next performed *in utero* electroporation experiments to determine whether forced expression or knockdown of *Eurl* influenced β-catenin signalling within the embryonic cortex. To achieve this, we electroporated the 8XTOPFLASHd2EGFP reporter construct together with *Eurl* expression vector or targeting siRNAs and assessed the proportion of GFP-expressing cells 24 h after electroporation. An RFP expression plasmid was co-delivered to cells to mark their successful co-electroporation. As shown in Fig. 8D,E, forced expression of *Eurl* led to an increase in β-catenin signalling in cells of the VZ compared to control, represented as an increased proportion of RFP+ cells which co-express GFP. In contrast, treatment with *Eurl* siRNAs led to a significant decrease in GFP-labelled cells, suggesting a reduction in Wnt/β-catenin signalling. Thus, our results indicate that alterations to *Eurl* expression result in concomitant changes to β-catenin signalling during cerebral cortical development.

## Discussion

In this study, we have characterised the expression pattern and neuronal function for EURL during mammalian cerebral cortex development. We find that EURL is expressed in the developing cerebral cortex of mice and humans, and its mRNA levels are altered in Down Syndrome brain tissue. We also find that perturbations to *Eurl* impair the long-term positioning and dendritic spine densities of cortical projection neurons. We provide a mechanistic link between *Eurl* expression and modulation of β-catenin signalling, and suggest how this axis might be crucially disrupted in circumstances of altered *Eurl* levels, leading to abnormal cortical organisation.

Our expression studies reveal widespread EURL expression within precursor cells and postmitotic neurons of both mouse and human fetal cortex. Such patterns of gene expression could reflect changing requirements for EURL during development. Additionally, our *EURL* mRNA expression analysis revealed diverse temporal patterns of gene expression in the dorsolateral prefrontal cortex, primary visual cortex and cerebellum. While these patterns of gene expression could reflect alterations in cellular requirements for *EURL* as the brain ages, we believe that the dynamic regulation of *EURL* is critical to its functions in neuronal development. In support of this hypothesis, our functional studies with mice demonstrate that suppression of *Eurl* leads to a decrease in neuroprogenitor proliferation, while forced expression leads to an increase in cell proliferation. This suggests that *Eurl* can exert its effects on target neurons in a concentration-sensitive manner (Supplementary Fig. 3A). Presently, it is unclear as to whether elevated expression of *Eurl* will lead to a significant alteration in brain size, since we found that while forced expression of *Eurl* led to an elevation in cortical progenitors co-labelled with Pax6 and Tbr2, together with increases to Ki67 and pHH3 expression in electroporated cells. Interestingly, we did not observe a concomitant reduction in TUJ1-expressing cells, hence our results suggest that forced expression of *Eurl* disrupts the neurogenic cell cycle and alters the pool of cortical progenitors. It remains to be clarified as to whether these effects of *Eurl* overexpression in progenitor cells are a cause or consequence of altered β-catenin signalling.

In contrast to its effects on progenitor cells, we find that both knockdown and forced expression of *Eurl* impairs the development of cortical projection neurons. Thus, it would appear that a permissive dose of *Eurl* is necessary for neuronal differentiation, with too much or too little both resulting in their defective development. It would also appear that *Eurl* has different dosage effects on neuroprogenitors versus postmitotic neurons (Supplementary Fig. 3B). These findings are consistent with the notion that gene dosage of *EURL* is an important facet of normal cortical development.

Our current understanding of EURL function is limited, thus our demonstration of its interaction with CCDC85B, a modulator of β-catenin signalling, is significant. Notably, our *in vitro* and *in vivo* studies provide the first supportive evidence that β-catenin signalling is modulated by EURL, and our data points to a mechanism in which EURL influences intracellular β-catenin protein levels to exert its effects on cell proliferation. During cortical development, disruptions to β-catenin signalling have been found to alter progenitor proliferation and neurogenesis^34^,^35^,^36^,^37^. Furthermore, β-catenin signalling is critical to the terminal differentiation of cortical projection neurons as well as their dendritic spine densities^35^,^38^,^39^,^40^,^41^. Our results would therefore suggest that EURL modulates β-catenin signalling to control the production and maturation of neurons during cerebral cortex development.

Within the human fetal cortex, EURL is detected in cells of the germinal zone, thus implicating a functional role in the genesis and maturation of cerebral cortical neurons. Guided by our findings in mice which show that alterations to *Eurl* disrupt neuronal development, we predict that altered *EURL* dosage likely contributes to neurodevelopmental abnormalities in human cortical development. Based on our results, we might expect that elevated expression of *EURL* (as a consequence of an extra copy of the gene in Down-Syndrome subjects) would enhance β-catenin signalling and increase brain growth. However, the opposite is found, since microcephaly is strongly associated with Down Syndrome. Such observations reflect the complexity in the molecular mechanisms which underlie *HSA21*-related disorders. Regardless, our studies provide additional evidence that alterations to the expression levels for huCHR21q21.1 candidate genes, such as *EURL*, have a direct consequence on nervous system development.

The study of EURL in mouse models of Down Syndrome is currently limited by the observation that this gene is not represented within the trisomic regions of HSA21 modelled in Ts65Dn, Ts1Cje, Ts1Rhr and Tc1 strains of mice (data not shown). Thus, our electroporation studies with mice crucially provide the first evidence that disruptions to *Eurl* expression result in alterations to cortical progenitor proliferation and neurogenesis. Furthermore, our studies draw a tentative link between alterations to *EURL* expression, disruptions to β-catenin signalling and brain disorder. Since patients diagnosed with Down Syndrome show clear evidence of abnormal neuronal development, including reduced brain volumes^6^, disorganised cortical layers indicative of aberrant neuronal positioning^7^, and reduced dendritic spine densities on cortical neurons^42^; we suggest that altered *EURL* dosage contributes to such neurological deficits in Down Syndrome.

## Methods

### DNA plasmids and Antibodies

Mammalian expression vectors pCIG2 (modified to comprise an N-terminal FLAG tag) and pcDNA3 (modified to comprise an N-terminal FLAG or HA epitope tag) were digested with EcoRI and treated with shrimp alkaline phosphatase (Promega, Australia) before ligation to mouse *Eurl* cDNA amplified by PCR using primers which encoded flanking MfeI restriction sites, together with a template (IMAGE:3966397 also known as D16Ertd472e, Genbank Accession Number BC019957). A similar strategy was employed to clone mouse *Ccdc85b* cDNA from IMAGE:5710206 (Genbank Accession Number BC058411). All constructs were sequenced to confirm directionality and nucleotide identity. For RNA interference studies, *Eurl* siRNAs (Catalogue number J-043562, Dharmacon, Thermofisher Scientific) and control siRNAs (D-001810-10-05) were resuspended in RNAse-free water and stored in aliquots before use. This *Eurl* siRNA mix comprises 3 species of siRNAs which target the 3′UTR, and one species which targets the coding sequence. For RNAi studies employing an shRNA vector, a hairpin cDNA sequence comprising *Eurl* targeting sequence 5′-CCTCCAAGTGGGACGGACA-3′ was cloned in pSilCaggs expression vector^43^. For *Eurl* siRNA rescue experiments, a cDNA encoding silent mutations to the abovementioned *Eurl* cDNA sequence (resulting in the cDNA sequence 5′-CCTCCAAGTGGGGAGAACA-3′; the mutated nucleotides are underlined) was cloned by PCR and inserted into pCIG2. Additionally, the GFP expression cassette from pCIG2 was also excised from *Eurl* constructs utilised in reporter studies in conjunction with 8XTOPFLASHd2EGFP reporter. We thank Dr Christophe Marcelle (EMBL Australia, Monash University) for providing the 8XTOPFLASHd2EGFP construct. Primary antibodies used for immunostaining analysis include chicken polyclonal antibody to GFP (Abcam, ab13970, 1:700), rabbit polyclonal antibody to EURL (sc-83610, Santa Cruz Biotechnologies, 1:500), mouse monoclonal anti-Eurl (Abmart), rabbit polyclonal antii-Ki67 (NCL-Ki67p, Leica, 1:1000), pHH3(ser10) (06-570, Merck Millipore, 1:1000), mouse anti TUJ1 (Covance, MMS-435P, 1:1000), mouse anti-neuronal nuclei (NeuN; MAB377, Merck Millipore), rat anti-CTIP2 (ab18465, Abcam), rabbit polyclonal antibody to GFP (Invitrogen, A6455, 1:1000). Alexa fluor secondary antibodies include goat anti- chicken IgG (Invitrogen, A11039, 1:700), goat anti-mouse (Invitrogen, A11031, 1:800), and goat anti-rabbit IgG (Invitrogen, A6455, 1:1000). The nuclei of cells were visualised with DAPI.

### Human fetal brain tissue for immunostaining studies

Human fetal telencephalon tissue samples were processed individually as previously described^44^. Specimens between 16–19 weeks gestation (WG) were obtained from second trimester human fetuses under approval from the Human Ethics Committee of the University of Sydney (HREC approval numbers 2006/9060 and 2012/15186) and the Melbourne Health Human Research Ethics Committee with informed consent. This work was carried out in accordance with the NHMRC National Statement on Ethical Conduct in human research. Brain tissue was dissected and transported in ice-cold HEPES-buffered MEM (Invitrogen, Carlsbad, CA) and fixed in 4% paraformaldehyde (PFA) (w/v) in 0.1M phosphate buffer (PB) for 1–9h at 41 °C. Fixed tissue was cryoprotected in 20% sucrose in PB, embedded in OCT compound (Tissue Tek) at −80 °C and cryosectioned at 12 μm with a Cryostat (Leica CM3050).

### Human tissue gene expression analysis

Human brain specimens were obtained from tissue collections at the Department of Neurobiology, Yale School of Medicine, and the University of Maryland Brain and Tissue Bank (Baltimore, MD). Brain tissues were collected after obtaining appropriate informed consent (from parent or next of kin). All procedures for the use of these tissue samples were approved by the Institutional review boards of the Yale School of Medicine and the University of Maryland, in accordance with regulations and ethical guidelines for the research use of human brain tissue set out by the National Institutes of Health (NIH, USA), and the World Medical Association (WMA) Declaration of Helsinki. All available non-identifying information was recorded for each tissue specimen. Trisomy21 was confirmed by karyotyping and/or Illumina Omni-2.5 million SNP arrays. Down syndrome samples and Control samples were matched on the basis of age, sex, race, post mortem interval, and RNA integrity. Affymetrix Human 1.0 ST arrays and the Affymetrix GeneChip platform were used for transcriptome analysis, as previously described^45^. Affymetrix exon array raw data (.CEL files) were normalized by Partek Genomics Suite version 6.6 to generate probeset-level (exon-level) and transcript cluster (gene-level) intensities. The expression level of a probe set was estimated by averaging the intensities of all core probe sets within the exon. We applied the following default Partek settings: RMA background correction, exclusion of probes containing SNPs, quantile normalization, mean probe set summarization, and log2-transformation. Only core probe sets defined by Affymetrix were included for calculations, as these core probe sets are of reliable sequence annotation. The expression level of a gene (transcript cluster) was estimated using the median of all exons within the gene.

### Animals

Mice (C57BL/6J) were maintained within the animal facilities at Monash University. Female mice of at least 6 weeks of age were utilised for timed-matings. All animal procedures are approved by the Monash University Animal Ethics Committee (MARP/2012/069) and compliant with guidelines provided by the National Health and Medical Research Council of Australia.

### *In utero* electroporation

*In utero* electroporation experiments with time-mated E14.5 pregnant female mice were performed as previously described^46^. High quality, low endotoxin plasmid preparations (Qiagen) of DNA vectors were injected at 1 μg/μl for each plasmid, together with Fast Green (0.05%, Sigma). For RNAi experiments, Dharmacon siRNA targeting pools for *Eurl* were injected at 10 μM concentration together with GFP expression plasmid at 1 μg/μl concentration. Following recovery from *in utero* electroporation and recovery from surgery, embryos were collected 36 hours or 48 hours later, as indicated for embryonic mouse brain studies. Mice were sacrificed by cervical dislocation, and the embryonic brains were harvested by dissection in cold PBS, preserved for tissue fixation with 4%paraformaldehyde/PBS, washing with PBS followed by sucrose (20% in PBS) penetration, OCT embedding and cryosectioning (16 μm) to prepare free-floating sections which were then subject to fluorescence immunostaining. In studies with postnatal mice, electroporated pups were born and raised until postnatal day P17. At the time of tissue collection, pups were anaesthetized before transcardial perfusion was performed to preserve brain tissue for subsequent analysis as 16 μm immobilised on glass slides, as previously described^46^. Immunostaining was performed as previously described^47^. Images of brain sections were captured on an epifluorescence microscope (Olympus) equipped with a CCD camera (SPOT), as well as using an Abrio C1 Upright confocal laser microscope (Nikon). Subdivisions of the embryonic cortex (VZ/SVZ, IZ and CP) were identified based on cell density as visualised with DAPI (4′6-Diamidino-2-Phenylindole) staining, as previously described^46^,^47^. Cell counting was performed by operators blinded to the experimental condition on representative fields of sections of electroporated brains using ImageJ software. For dendritic spine density studies, images of layer II/III neurons were generated as maximum intensity projections of stacked confocal images acquired at X60 magnification with 1.832x zoom using an oil immersion lens. Quantitation of dendritic spine protrusions was performed along the length of the main apical dendrite by visual inspection. The average length of apical dendrites measured in this study across all conditions was 104.3 + 2.74 μm (mean + SEM).

### Cell Culture, Western blotting and immunoprecipitation

Human Embryonic Kidney (HEK293T) and HeLa tumour cells, as well as mouse neuroblastoma (Neuro2A) and embryonic carcinoma (P19) cells were cultured in Dulbecco’s modified Eagle’s medium (Gibco 10313) supplemented with 10% heat-inactivated fetal bovine serum (Thermo Fisher HYC15-010.02), 2 mM L-glutamine (Gibco 25030), 20 units/ml of penicillin and streptomycin (Gibco 15140) under humidified air containing 5% CO2 at 37 °C. Media for Neuro2A cells was supplemented with 1% Non-essential Amino acids (Gibco 10370-021). Transfections were performed using equal quantities of expression plasmids for each condition. Immunoprecipitation and Western blotting analysis was performed with antibodies to FLAG (F1804, Sigma Aldrich), myc (Ab9106, Abcam), actin (A5441, Sigma Aldrich), β-tubulin (T0198, Sigma Aldrich) together with appropriate fluorescent secondary antibodies, as described^48^. Immunoblot signals were resolved detected with an Odyssey® infrared imaging system (Li-Cor 9201-02) for analysis.

### Statistical analysis

Data are presented as means ± standard error. Two-tailed unpaired Student’s t-test was used for analysing morphology and spine density between control and *Eurl* shRNA treatment. One-way ANOVA with Bonferroni post-hoc test was used for comparing the data between control and *Eurl* siRNA treatment, *Eurl* overexpression, and *Eurl* siRNA co-treated with *Eurl* expression construct. *, ** and *** indicates P < 0.05, P < 0.01, P < 0.001 respectively. A P-value of <0.05 was considered statistically significant.

## Additional Information

**How to cite this article**: Li, S. S. *et al*. The HSA21 gene *EURL*/*C21ORF91* controls neurogenesis within the cerebral cortex and is implicated in the pathogenesis of Down Syndrome. *Sci. Rep.* **6**, 29514; doi: 10.1038/srep29514 (2016).

## Supplementary Material

Supplementary Information

## References

[b1] Heng J. I., Chariot A. & Nguyen L. Molecular layers underlying cytoskeletal remodelling during cortical development. Trends Neurosci 33, 38–47, 10.1016/j.tins.2009.09.003 (2010). 19837469

[b2] Kriegstein A. R. & Noctor S. C. Patterns of neuronal migration in the embryonic cortex. Trends Neurosci 27, 392–399, 10.1016/j.tins.2004.05.001 S0166223604001547 (2004). 15219738

[b3] Leventer R. J., Guerrini R. & Dobyns W. B. Malformations of cortical development and epilepsy. Dialogues Clin Neurosci 10, 47–62 (2008). 18472484 10.31887/DCNS.2008.10.1/rjleventer PMC3181860

[b4] Haydar T. F. & Reeves R. H. Trisomy 21 and early brain development. Trends Neurosci 35, 81–91, 10.1016/j.tins.2011.11.001 (2012). 22169531 PMC3273608

[b5] Rachidi M. & Lopes C. Molecular and cellular mechanisms elucidating neurocognitive basis of functional impairments associated with intellectual disability in Down syndrome. Am. J. Intellect. Dev. Disabil. 115, 83–112, 10.1352/1944-7558-115.2.83 (2010). 20441388

[b6] Pinter J. D., Eliez S., Schmitt J. E., Capone G. T. & Reiss A. L. Neuroanatomy of Down’s syndrome: a high-resolution MRI study. The Am. J. Psychiat. 158, 1659–1665 (2001). 11578999 10.1176/appi.ajp.158.10.1659

[b7] Golden J. A. & Hyman B. T. Development of the superior temporal neocortex is anomalous in trisomy 21. J. Neuropath. Exp. Neur. 53, 513–520 (1994). 8083693 10.1097/00005072-199409000-00011

[b8] Marin-Padilla M. Pyramidal cell abnormalities in the motor cortex of a child with Down’s syndrome. A Golgi study. J. Comp. Neurol. 167, 63–81, 10.1002/cne.901670105 (1976). 131810

[b9] Petit T. L., LeBoutillier J. C., Alfano D. P. & Becker L. E. Synaptic development in the human fetus: a morphometric analysis of normal and Down’s syndrome neocortex. Exp. Neurol. 83, 13–23 (1984). 6228436 10.1016/0014-4886(84)90041-4

[b10] Takashima S., Becker L. E., Armstrong D. L. & Chan F. Abnormal neuronal development in the visual cortex of the human fetus and infant with down’s syndrome. A quantitative and qualitative Golgi study. Brain. Res. 225, 1–21 (1981). 6457667 10.1016/0006-8993(81)90314-0

[b11] Weitzdoerfer R., Dierssen M., Fountoulakis M. & Lubec G. Fetal life in Down syndrome starts with normal neuronal density but impaired dendritic spines and synaptosomal structure. J Neur. Tr. S. 59–70, 10.1007/978-3-7091-6262-0_5 (2001). 11771761

[b12] Hwang S. W. & Jea A. A review of the neurological and neurosurgical implications of Down syndrome in children. Clin. Pediatr. 52, 845–856, 10.1177/0009922813491311 (2013). 23743011

[b13] Stafstrom C. E., Patxot O. F., Gilmore H. E. & Wisniewski K. E. Seizures in children with Down syndrome: etiology, characteristics and outcome. Dev. Med. Child. Neurol. 33, 191–200 (1991). 1827417 10.1111/j.1469-8749.1991.tb05108.x

[b14] Chakrabarti L. . Olig1 and Olig2 triplication causes developmental brain defects in Down syndrome. Nat. Neurosci. 13, 927–934, 10.1038/nn.2600 (2010). 20639873 PMC3249618

[b15] Maynard K. R. & Stein E. DSCAM contributes to dendrite arborization and spine formation in the developing cerebral cortex. J. Neurosci. 32, 16637–16650, 10.1523/JNEUROSCI.2811-12.2012 (2012). 23175819 PMC6621782

[b16] Yabut O., Domogauer J. & D’Arcangelo G. Dyrk1A overexpression inhibits proliferation and induces premature neuronal differentiation of neural progenitor cells. J. Neurosci. 30, 4004–4014, 10.1523/JNEUROSCI.4711-09.2010 (2010). 20237271 PMC3842457

[b17] Zhang L., Huang Y., Chen J. Y., Ding Y. Q. & Song N. N. DSCAM and DSCAML1 regulate the radial migration and callosal projection in developing cerebral cortex. Brain. Res. 1594, 61–70, 10.1016/j.brainres.2014.10.060 (2015). 25451118

[b18] Korbel J. O. . The genetic architecture of Down syndrome phenotypes revealed by high-resolution analysis of human segmental trisomies. Proc. Natl. Acad. Sci. USA 106, 12031–12036, 10.1073/pnas.0813248106 (2009). 19597142 PMC2709665

[b19] Rost I. . Tetrasomy 21pter– >q21.2 in a male infant without typical Down’s syndrome dysmorphic features but moderate mental retardation. J. Med. Genet. 41, e26 (2004). 14985397 10.1136/jmg.2003.011833 PMC1735700

[b20] Slavotinek A. M. . Partial tetrasomy 21 in a male infant. J. Med. Genet. 37, E30 (2000). 11015462 10.1136/jmg.37.10.e30 PMC1757147

[b21] Godbout R., Andison R., Katyal S. & Bisgrove D. A. Isolation of a novel cDNA enriched in the undifferentiated chick retina and lens. Dev. Dyn. 227, 409–415, 10.1002/dvdy.10310 (2003). 12815627

[b22] Ait Yahya-Graison E. . Classification of human chromosome 21 gene-expression variations in Down syndrome: impact on disease phenotypes. Am J. Hum. Genet. 81, 475–491, 10.1086/520000 (2007). 17701894 PMC1950826

[b23] Prandini P. . Natural gene-expression variation in Down syndrome modulates the outcome of gene-dosage imbalance. Am. J. Hum. Genet. 81, 252–263, 10.1086/519248 (2007). 17668376 PMC1950802

[b24] Visel A., Thaller C. & Eichele G. GenePaint.org: an atlas of gene expression patterns in the mouse embryo. Nucleic. Acids. Res. 32, D552–D556, 10.1093/nar/gkh029 (2004). 14681479 PMC308763

[b25] Consortium F. . A promoter-level mammalian expression atlas. Nature 507, 462–470, 10.1038/nature13182 (2014). 24670764 PMC4529748

[b26] Olmos-Serrano J. L. . Down Syndrome Developmental Brain Transcriptome Reveals Defective Oligodendrocyte Differentiation and Myelination. Neuron 89, 1208–1222, doi.i: 10.1016/j.neuron.2016.01.042 (2016). 26924435 PMC4795969

[b27] Merot Y., Retaux S. & Heng J. I. Molecular mechanisms of projection neuron production and maturation in the developing cerebral cortex. Semin. Cell. Dev. Biol. 20, 726–734, 10.1016/j.semcdb.2009.04.003 (2009). 19442543

[b28] Nguyen L. . p27kip1 independently promotes neuronal differentiation and migration in the cerebral cortex. Gene. Dev. 20, 1511–1524, 10.1101/gad.377106 (2006). 16705040 PMC1475763

[b29] Chatr-Aryamontri A. . The BioGRID interaction database: 2015 update. Nucleic. Acids. Res. 43, D470–D478, 10.1093/nar/gku1204 (2015). 25428363 PMC4383984

[b30] Markham N. O. . DIPA-family coiled-coils bind conserved isoform-specific head domain of p120-catenin family: potential roles in hydrocephalus and heterotopia. Mol. Biol. Cell. 25, 2592–2603, 10.1091/mbc.E13-08-0492 (2014). 25009281 PMC4148249

[b31] Iwai A. . Coiled-coil domain containing 85B suppresses the beta-catenin activity in a p53-dependent manner. Oncogene. 27, 1520–1526, 10.1038/sj.onc.1210801 (2008). 17873903

[b32] Rios A. C., Serralbo O., Salgado D. & Marcelle C. Neural crest regulates myogenesis through the transient activation of NOTCH. Nature. 473, 532–535, 10.1038/nature09970 (2011). 21572437

[b33] Klein P. S. & Melton D. A. A molecular mechanism for the effect of lithium on development. Proc. Natl. Acad. Sci. USA 93, 8455–8459 (1996). 8710892 10.1073/pnas.93.16.8455 PMC38692

[b34] Chenn A. & Walsh C. A. Regulation of cerebral cortical size by control of cell cycle exit in neural precursors. Science 297, 365–369, 10.1126/science.1074192 (2002). 12130776

[b35] Mutch C. A., Funatsu N., Monuki E. S. & Chenn A. Beta-catenin signaling levels in progenitors influence the laminar cell fates of projection neurons. J. Neurosci. 29, 13710–13719, 10.1523/JNEUROSCI.3022-09.2009 (2009). 19864583 PMC2786782

[b36] Singh K. K. . Common DISC1 polymorphisms disrupt Wnt/GSK3beta signaling and brain development. Neuron. 72, 545–558, 10.1016/j.neuron.2011.09.030 (2011). 22099458 PMC3387684

[b37] Wrobel C. N., Mutch C. A., Swaminathan S., Taketo M. M. & Chenn A. Persistent expression of stabilized beta-catenin delays maturation of radial glial cells into intermediate progenitors. Dev. Biol. 309, 285–297, 10.1016/j.ydbio.2007.07.013 (2007). 17706960 PMC2083700

[b38] Murase S., Mosser E. & Schuman E. M. Depolarization drives beta-Catenin into neuronal spines promoting changes in synaptic structure and function. Neuron 35, 91–105 (2002). 12123611 10.1016/s0896-6273(02)00764-x

[b39] Okuda T., Yu L. M., Cingolani L. A., Kemler R. & Goda Y. beta-Catenin regulates excitatory postsynaptic strength at hippocampal synapses. Proc. Natl. Acad. Sci. USA 104, 13479–13484, 10.1073/pnas.0702334104 (2007). 17679699 PMC1948936

[b40] Peng Y. R. . Coordinated changes in dendritic arborization and synaptic strength during neural circuit development. Neuron 61, 71–84, 10.1016/j.neuron.2008.11.015 (2009). 19146814 PMC2713111

[b41] Yu X. & Malenka R. C. Beta-catenin is critical for dendritic morphogenesis. Nat. Neurosci. 6, 1169–1177, 10.1038/nn1132 (2003). 14528308

[b42] Ferrer I. & Gullotta F. Down’s syndrome and Alzheimer’s disease: dendritic spine counts in the hippocampus. Acta. Neuropathol. 79, 680–685 (1990). 2141748 10.1007/BF00294247

[b43] Bron R. . Boundary cap cells constrain spinal motor neuron somal migration at motor exit points by a semaphorin-plexin mechanism. Neural. Dev. 2, 21, 10.1186/1749-8104-2-21 (2007). 17971221 PMC2131750

[b44] Weible M. W. 2nd & Chan-Ling T. Phenotypic characterization of neural stem cells from human fetal spinal cord: synergistic effect of LIF and BMP4 to generate astrocytes. Glia 55, 1156–1168, 10.1002/glia.20539 (2007). 17597119

[b45] Kang H. J. . Spatio-temporal transcriptome of the human brain. Nature 478, 483–489, 10.1038/nature10523 (2011). 22031440 PMC3566780

[b46] Breuss M. . Mutations in the beta-tubulin gene TUBB5 cause microcephaly with structural brain abnormalities. Cell reports 2, 1554–1562, 10.1016/j.celrep.2012.11.017 (2012). 23246003 PMC3595605

[b47] Heng J. I. . Neurogenin 2 controls cortical neuron migration through regulation of Rnd2. Nature 455, 114–118, 10.1038/nature07198 (2008). 18690213

[b48] Gladwyn-Ng I. E. . Bacurd2 is a novel interacting partner to Rnd2 which controls radial migration within the developing mammalian cerebral cortex. Neural. Dev. 10, 9, 10.1186/s13064-015-0032-z (2015). 25888806 PMC4433056

