# Supplementary material for: The HSA21 gene EURL/C21ORF91 controls neurogenesis within the cerebral cortex and is implicated in the pathogenesis of Down Syndrome
Source: Sci Rep. 2016 Jul 11;6:29514. doi: 10.1038/srep29514 (PMC4941730; doi:10.1038/srep29514)
Supplement: Supplementary Information [file srep29514-s1.pdf]

The HSA21 gene *EURL/C21ORF91* controls neurogenesis within the cerebral cortex and is implicated in the pathogenesis of Down Syndrome

Shan Shan Li[1,8], Zhengdong Qu[1,8], Matilda Haas[1], Linh Ngo[1, 2], You Jeong Heo[3], Hyo Jung Kang[3,4], Joanne Maria Britto[5], Hayley Daniella Cullen[2], Hannah Kate Vanyai[2], Seong-Seng Tan[5], Tailoi Chan-Ling[6], Jenny Margaret Gunnersen[5,7], Julian Ik-Tsen Heng[1,2,*].

Supplementary Material (3 Supplementary Figures)

[1]EMBL Australia, The Australian Regenerative Medicine Institute, Monash University, Clayton, Victoria 3800, Australia

[2]The Harry Perkins Institute of Medical Research, QEII Medical Centre, Nedlands and Centre for Medical Research, The University of Western Australia, Crawley, Western Australia, 6009, Australia

[3]Department of Life Science, Chung-Ang University, Seoul, Korea

[4]Department of Neurobiology, Yale School of Medicine, New Haven, CT 06510, USA

[5]The Florey Institute of Neuroscience and Mental Health, Parkville, Victoria 3010, Australia

[6]Discipline of Anatomy, School of Medical Sciences, Bosch Institute, The University of Sydney, Sydney, New South Wales, Australia

[7]The Anatomy and Neuroscience Department, University of Melbourne, Parkville, Victoria 3010, Australia

[8]these authors contributed equally to this work

[*]Corresponding author: julian.heng@perkins.uwa.edu.au

Supplementary Figure 1

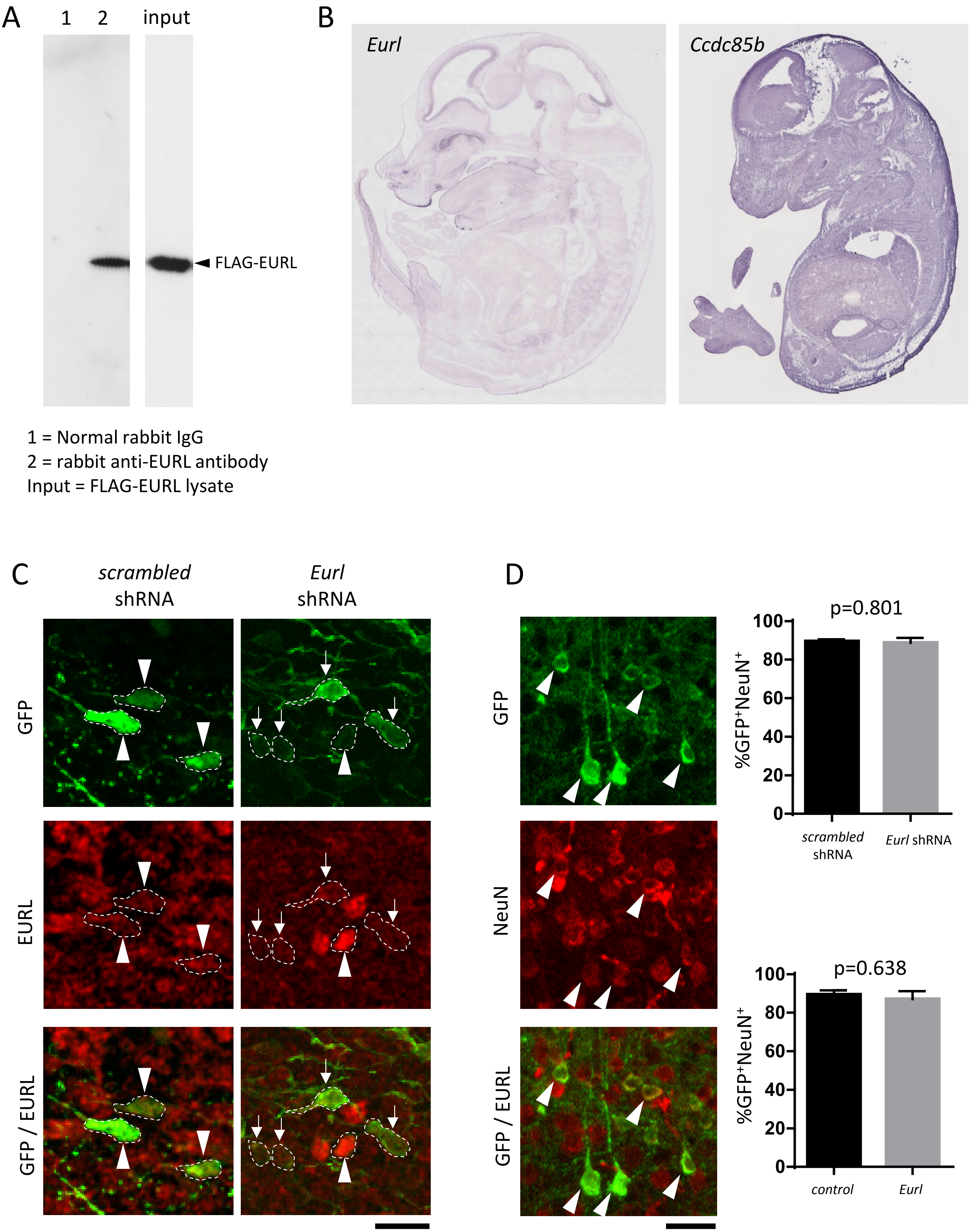

Supplementary Figure 2

A

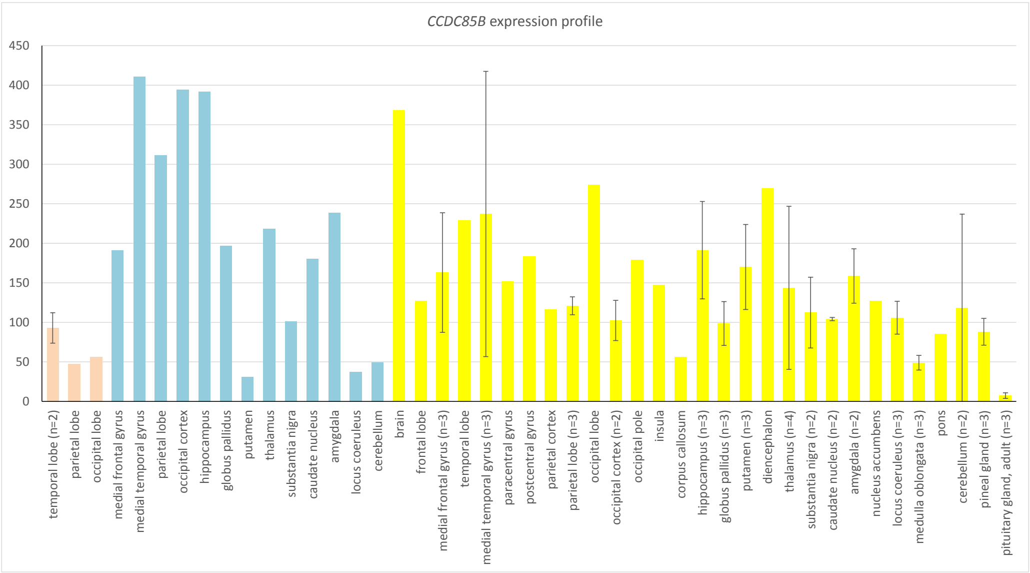

B

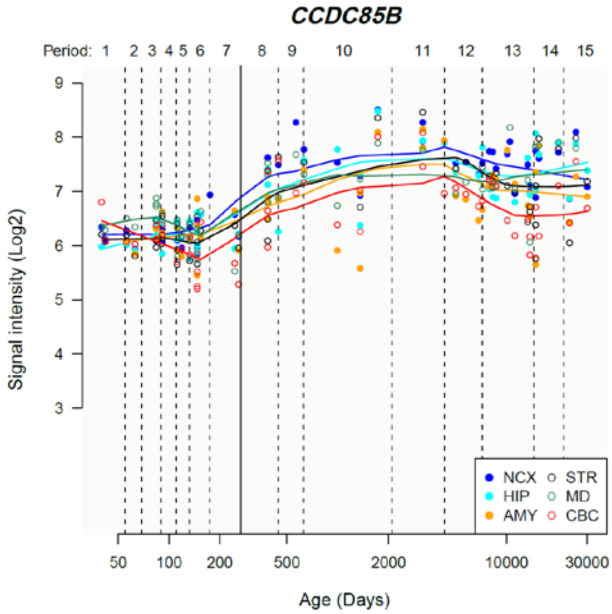

C

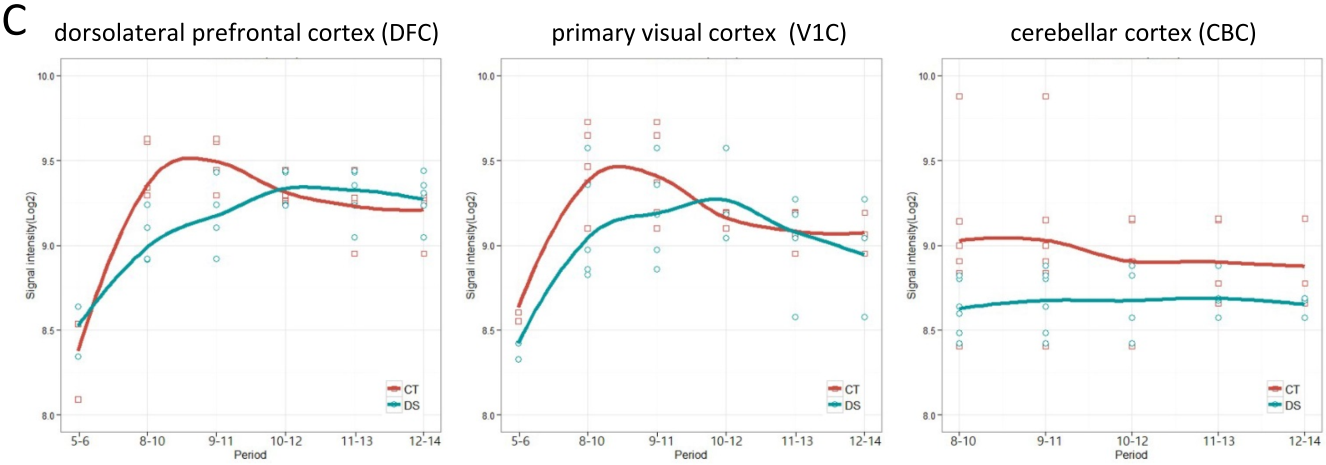

Age ranges for Periods

5..6	16wg≤ X < 23wg
8..10	Births≤ X < 6 yrs
9..11	6 months≤ X < 12 yrs
10..12	1 yrs≤ X < 20 yrs
11..13	6 yrs ≤ X < 40 yrs
12..14	12 yrs ≤ X < 60 yrs

Supplementary Figure 3

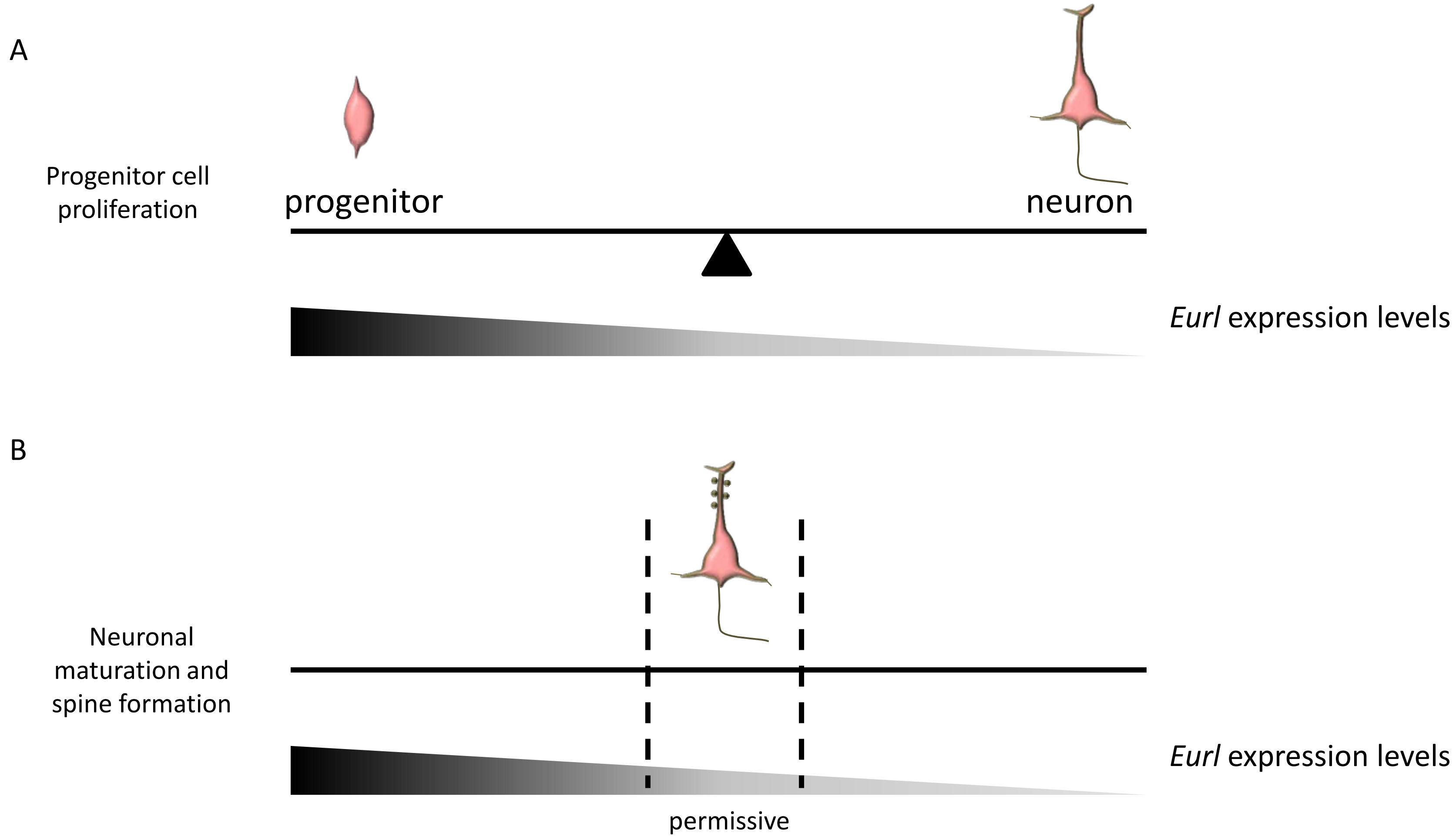

Legends to Supplementary Figures

Supplementary Figure 1. (***A***) The EURL antibody immunoprecipitates FLAG-tagged EURL from transiently transfected cell lysates, while a naïve antibody species fails to immunoprecipitate this product. Signals for immunoprecipitation and input lanes are from the same blot sampled at different exposure times. (***B***) Images of *in situ* hybridisation signals for *Eurl* and *Ccdc85b* on sagittal sections of E14.5 mouse embryos from the expression atlas GenePaint, with details of cRNA probes and NBT/BCIP colorimetric detection as reported[24]. Prominent *Eurl* signal is detected in the VZ of the embryonic cortex. (***C***) Immunolabelling of EURL knockdown cells following electroporation with targeting shRNAs. *In utero* electroporation was performed to deliver shRNA plasmids encoding a scrambled (non-targeting) sequence or a *Eurl* targeting sequence into E14.5 embryonic mouse brains (see Methods). Successfully electroporated cells were co-labelled by GFP expression which is conferred by an expression cassette within the shRNA plasmid. Cells within the intermediate zone (IZ) of the E17.5 mouse embryonic cortex were imaged by confocal microscopy. As shown, while scrambled shRNA-treated cells are immunostained for EURL (indicated with arrowheads), *Eurl* shRNA-treated cells show reduced EURL immunoreactivity (arrows point to EURL-deficient cell bodies), although EURL immunoreactivity persists in some cells (indicated by arrowhead). (***D***) Representative confocal image of GFP-electroporated cells within the P17 cortex which co-label with the pan-neuronal marker NeuN. There is no significant difference in the proportion of NeuN-immunoreactive cells following knockdown (>740 cells counted from at least 4 independent samples per condition, unpaired *t*-tests, two-tailed) or overexpression (>670 cells counted from at least 4 independent samples per condition, unpaired *t*-tests, two-tailed) of *Eurl*. Scale bars represents 20μm.

Supplementary Figure 2. Expression patterns for *CCDC85B* mRNA in human brain. (***A***) A survey of *CCDC85B* mRNA expression levels in human brain tissue samples from fetal (pink bars), newborn (light blue) and adult (yellow bars) generated through Capped Analysis of Gene Expression (CAGE) in FANTOM5 (24). Data was collected from identical tissue libraries used to derive *EURL*/*C21ORF91* mRNA expression data (Figure 2), and plotted as average $\pm$ standard deviation. (***B***) Temporal analysis of *CCDC85B* mRNA in the human brain reveals a dynamic expression pattern within the neocortex (NCX), striatum (STR), hippocampus (HIP), mediodorsal nucleus of the thalamus (MD), amygdala (AMY); and the cerebellar cortex (CBC). Similar trends in gene expression patterns across different brain tissues throughout life are observed, in which mRNA levels gradually increase in all tissues from birth until a decline is observed from approximately 27 years of age (10,000 days). The solid vertical line represents birth (defined as 280 days). (***C***) Analysis of *CCDC85B* mRNA expression in the dorsolateral prefrontal cortex (DFC), primary visual cortex (V1C) and cerebellar cortex (CBC) between Down Syndrome (DS, in green) patients and age-matched normotypic controls (CT, in red). There is a significant difference in the levels of *CCDC85B* mRNA within the V1C and the CBC (V1C, p=0.031, n=22; CBC, p=0.002, n=23; one-tailed *t*-test), but not the DFC (p=0.09, n=24; one-tailed *t*-test), indicating that alterations to *CCDC85B* expression levels within the brain underlie Down Syndrome.

Supplementary Figure 3. Summary scheme illustrating the consequences of altered *Eurl* levels on neuroprogenitor proliferation and neuronal differentiation.

(***A***) In this scheme, alterations to *Eurl* levels disrupt the proliferation of progenitor cells and the development of postmitotic neurons in different ways. Based on our results, we propose that excessive levels will sustain neuroprogenitor proliferation, while gene suppression results in cell cycle exit and neurogenesis. (***B***) In postmitotic neurons, a permissive dose of *Eurl* is essential to the long-term positioning and dendritic spine densities of cortical projection neurons, with too much or too little *Eurl* resulting in their altered cell positioning within the postnatal cortex, as well as reductions in the density of dendritic spines.
